# Could ChatGPT get an engineering degree? Evaluating higher education vulnerability to AI assistants

**DOI:** 10.1073/pnas.2414955121

**Published:** 2024-11-26

**Authors:** Beatriz Borges, Negar Foroutan, Deniz Bayazit, Anna Sotnikova, Syrielle Montariol, Tanya Nazaretzky, Mohammadreza Banaei, Alireza Sakhaeirad, Philippe Servant, Seyed Parsa Neshaei, Jibril Frej, Angelika Romanou, Gail Weiss, Sepideh Mamooler, Zeming Chen, Simin Fan, Silin Gao, Mete Ismayilzada, Debjit Paul, Philippe Schwaller, Sacha Friedli, Patrick Jermann, Tanja Käser, Antoine Bosselut, EPFL Grader Consortium, EPFL Data Consortium

**Affiliations:** ^a^École Polytechnique Fédérale de Lausanne (EPFL), Lausanne 1015, Switzerland

**Keywords:** LLM, education, generative AI, education vulnerability

## Abstract

AI assistants, such as ChatGPT, are being increasingly used by students in higher education institutions. While these tools provide opportunities for improved teaching and education, they also pose significant challenges for assessment and learning outcomes. We conceptualize these challenges through the lens of vulnerability, the potential for university assessments and learning outcomes to be impacted by student use of generative AI. We investigate the potential scale of this vulnerability by measuring the degree to which AI assistants can complete assessment questions in standard university-level Science, Technology, Engineering, and Mathematics (STEM) courses. Specifically, we compile a dataset of textual assessment questions from 50 courses at the École polytechnique fédérale de Lausanne (EPFL) and evaluate whether two AI assistants, GPT-3.5 and GPT-4 can adequately answer these questions. We use eight prompting strategies to produce responses and find that GPT-4 answers an average of 65.8% of questions correctly, and can even produce the correct answer across at least one prompting strategy for 85.1% of questions. When grouping courses in our dataset by degree program, these systems already pass the nonproject assessments of large numbers of core courses in various degree programs, posing risks to higher education accreditation that will be amplified as these models improve. Our results call for revising program-level assessment design in higher education in light of advances in generative AI.

## Significance

Universities primarily evaluate student learning through various course assessments. Our study demonstrates that AI assistants, such as ChatGPT, can answer at least 65.8% of examination questions correctly across 50 diverse courses in the technical and natural sciences. Our analysis demonstrates that these capabilities render many degree programs (and their teaching objectives) vulnerable to potential misuse of these systems. These findings call for attention to assessment design to mitigate the possibility that AI assistants could divert students from acquiring the knowledge and critical thinking skills that university programs are meant to instill.

ChatGPT, a system using a large language model (LLM), GPT-3.5, as its foundation, was released in November 2022 to broad adoption and fanfare, reaching 100M users in its first month of use and remaining in the public discourse to this day. As arguably the most hyped AI system to date, its release has prompted a continuing discussion of societal transformations likely to be induced by AI over the next years and decades. Potential changes in modern educational systems have remained a core topic in this discussion, with early reports highlighting the risk of these AI systems as tools that would allow students to succeed in university coursework without learning the relevant skills those courses are meant to teach. Despite this concern, there has yet to be a comprehensive empirical study of the potential impact of LLMs (and derivative tools) on the assessment methods that educational institutions use to evaluate students. A few studies have explored the interesting subtask of how well models perform on problems related to topics typically taught in many university courses and aggregated relevant question sets for this purpose (1–5). However, none of these works extrapolate these findings to assess the downstream impact of these tools on degree programs, where the risk of these technologies relative to their pedagogical benefits must actually be measured.

**Fig. 1.**
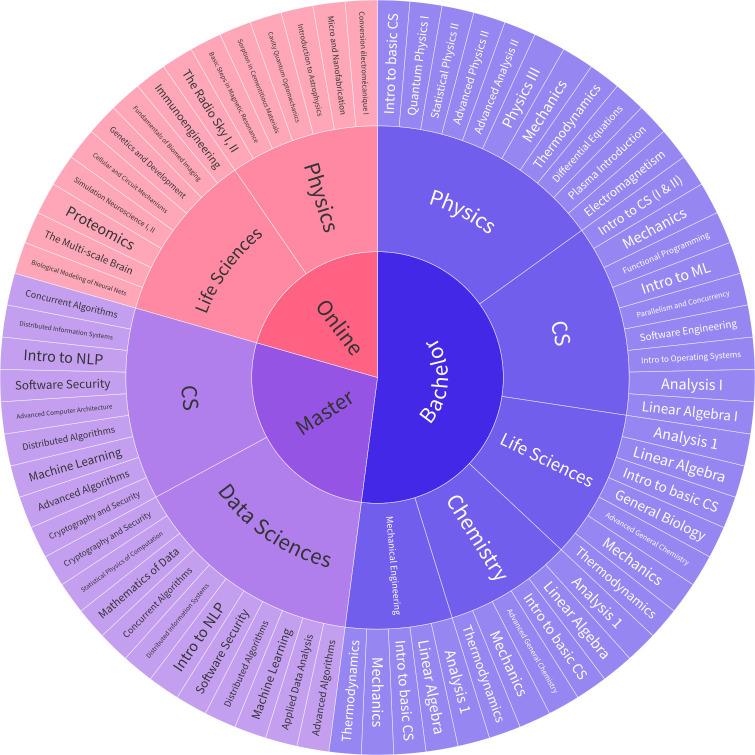
Overview of Courses. Courses represented in our dataset, grouped by program and degree. Courses may belong to multiple programs, in which case their partition is split into chunks of equal size, with one chunk assigned to each program.

In this work, we conduct a large-scale study across 50 courses from EPFL to measure the current performance of LLMs on higher education course assessments. The selected courses are sampled from 9 Bachelor’s, Master’s, and Online programs, covering between 33% and 66% of the required courses in these programs. From these courses, we compile a bilingual dataset (English and French) of 5,579 textual open-answer and multiple-choice questions (MCQ). All questions were extracted from real exams, assignments, and practical exercise sessions used to evaluate students in previous years. The course distribution is presented in Fig. 1, and the dataset statistics are shown in Table 1 (stratified by particular dataset attributes).

**Table 1.** Dataset statistics

	Category	Total questions
Level	Bachelor’s courses	2,147 (38.5%)
	Master’s courses	1,631 (29.2%)
	Online programs	1,801 (32.3%)
Language	English	3,933 (70.5%)
	French	1,646 (29.5%)
Question type	MCQ	3,460 (62%)
	Open-answer	2,119 (38%)

Using this dataset, we subsequently test two commonly used models, GPT-4 (6), the system widely considered to be the most performant (7) among public AI assistants^*^ and GPT-3.5 (8), a highly performant freely available system. We generate responses from these systems using a range of prompting strategies (9–16), each of which varies in complexity, but all of which could easily be applied by a lay practitioner with minimal training in prompt engineering (17). We evaluate these systems using both automatic and manual grading, where manual grading of open-answer questions is performed by the same faculty staff that designed these problems and who have experience grading student answers to them. Subsequently, we conduct a detailed analysis of the generated outputs and their assessment results, considering factors such as the number of courses that would be passed, their distribution across university programs, as well as the effects of the question difficulty and language.

Our results show that AI systems are relatively capable of answering questions used in university assessments. GPT-4 responds correctly to ∼65.8% of questions when aggregating responses across the different prompting strategies using a simple majority vote (i.e., a *knowledge-free* setting that assumes the user would use this tool with no subject knowledge). Furthermore, across the eight prompting strategies, GPT-4 can generate at least one correct response for 85.1% of questions (maximum performance), indicating even greater assessment vulnerability in a setting where a user may have enough subject knowledge to *select* a correct answer even if they cannot produce it. This performance is relatively stable across courses in various scientific disciplines, impacting courses regardless of their subject and size. Importantly, we find that these results indicate that large numbers of university degree programs are highly vulnerable to these tools, with the nonproject components of many core courses being passed in multiple degrees offered by our institution.

Finally, we observe that while these systems are capable of reaching passing grades in many university assessments, they struggle with more complex question types where students also tend to perform most poorly. Taken together, these results indicate a possibility that these systems could be used to achieve passing marks in university courses while circumventing the process by which students acquire basic domain knowledge and learn to extend it to more complex problems. We conclude with a discussion on mitigations to university assessment settings, an outlook on how university systems should adapt to the increased use of these tools, and a discussion of limitations of our study, specifically with respect to how it diverges from exact assessment and grading policies at our institution.

## Data Collection

We compile a dataset of assessment questions from 50 courses offered at our institution from both on-campus and online classes. Following data preprocessing and filtering steps, this dataset consists of a total bank of 5,579 textual multiple-choice (MCQ) and open-answer questions in both English and French. These questions span various levels (e.g., Bachelor, Master), and cover a broad spectrum of STEM disciplines, including Computer Science, Mathematics, Biology, Chemistry, Physics, and Material Sciences. Table 1 and Fig. 1 provide an overview of the dataset’s main statistics and the distribution of questions across different topics. Additionally, we have collected course-specific attributes such as the year when the course is first offered in our institution’s degree programs (e.g., *Master’s year 1*), the program designation (e.g., *Physics*), the language of instruction (e.g., *French*), and the average student enrollment over recent years. Finally, certain questions have been labeled by the instructor who designed the question with a subjective annotation of the question’s difficulty.

## Experimental Findings

In our study, we investigate the vulnerability of university programs to generative AI systems using our question bank of 5,579 evaluation questions from 50 courses. We consider two models, GPT-4 and GPT-3.5, selected due to their popularity and usage rates. GPT-4 is considered the most performant model among all publicly accessible LLMs but is only available through a premium subscription, impeding its use by many students. GPT-3.5 is a less performant alternative, but free to use. We generate responses to questions from these models using eight relatively easy-to-apply prompting methods (implementation details are described in *SI Appendix*, section 2). For multiple-choice questions, we assess whether a response is correct by comparing the selected choice with the annotated correct answer option. For open-response questions, we use GPT-4 to rate the quality of the response on a four-point scale: Correct, Mostly Correct, Mostly Incorrect, Incorrect, which we map to scores of 1, 0.66, 0.33, and 0, respectively, for calculating performance.^†^

### Can LLM Systems Pass University-Level Courses?.

We begin our analysis by assessing model performance in a setting where the user has zero knowledge about the question topic. In the simplest scenarios, where we use the most straightforward prompting strategies such as directly asking a question (zero-shot) or asking the model to provide a reasoning chain before answering the question (zero-shot chain-of-thought), GPT-4 achieves average accuracies of 55.9% and 65.1%, respectively. With a slightly more complex zero-knowledge strategy, such as majority voting over the eight answers generated by the different prompting strategies, they would receive a correct answer to 65.8% (on average) of questions using GPT-4 (and 52.2% using GPT-3.5). We observe that this performance trend holds regardless of the language of the assessment, with English exhibiting slightly better results than French. Further experimental results for assessments in English and French are detailed in *SI Appendix*, section 5.C.

**Fig. 2.**
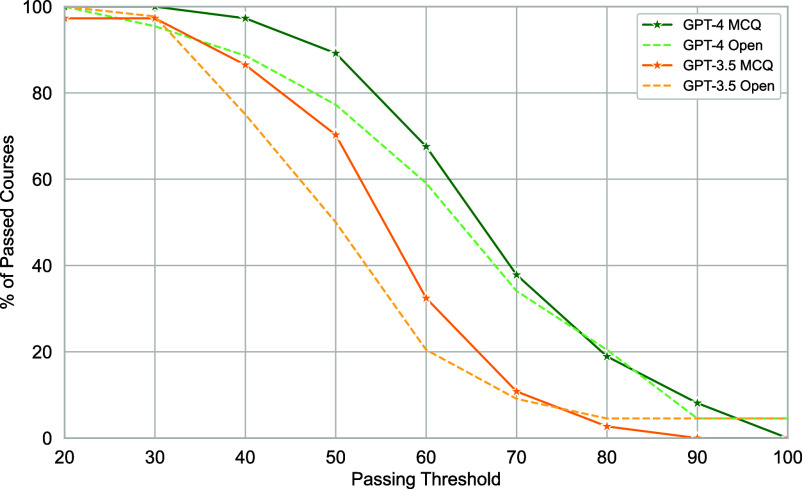
Course Pass Rate of Generative AI Assistants. Proportion of 50 courses that models successfully pass at various performance thresholds. Results are presented independently for multiple-choice (MCQ) and open-answer (Open) question types for both GPT-3.5 and GPT-4. Model responses are aggregated using the majority vote strategy.

Beyond overall performance across the question bank, Fig. 2 presents the proportion of passed courses for our sample of 50 courses based on varying passing thresholds. Alarmingly, GPT-4 can easily be used to reach a 50% performance threshold (which could be sufficient to pass many courses at various universities) for 89% of courses with MCQ-based evaluations and for 77% of courses with open-answer ones. While not performing quite as well, GPT-3.5, the freely available model, can reach a 50% threshold for 70% of courses with MCQ-based assessments and for 50% of courses with open-answer questions. As passing thresholds may not be set to 50% for all institutions, we vary this threshold and find that GPT-4 still passes 68% of courses at a 60% passing threshold, and 38% of courses at a 70% passing threshold for MCQ. Similar results are found for open-answer questions.

Our results suggest that users with no knowledge of a particular subject could solve enough questions to pass nonproject assessments in a majority of the courses in our dataset. While these observations make a compelling argument that AI assistants could potentially augment student learning as support tools, they simultaneously indicate a credible short-term risk to educational systems if institutions are not adapted to protect against the misuse of these technologies. Finally, we expect this problem to only grow worse over time, as continual model improvements in the years to come will make these tools even more performant in academic contexts.

### How Do These Results Affect University Programs?.

The average performance across courses demonstrates each course’s potential vulnerability to generative AI tools, which is particularly important if considerable portions of degree programs can be completed using these tools. To evaluate this program vulnerability, we aggregate the questions in our datasets according to the study programs in which they are core courses. Specifically, we include four program types: first-year Bachelor, Full Bachelor, Full Master, and Online courses. We separate the first year of the Bachelor’s degree because, at many institutions (including ours), the first year of the Bachelor’s may have a fairly standardized curriculum that serves a special purpose (e.g., replacing or complementing entrance exams). Full Bachelor’s and Master’s correspond to regular Bachelor’s and Master’s programs. We also include online courses, as official certifications can often be awarded for completing a sequence of these courses. For each program, our dataset contains a sample of courses that cover from 33% to 66% of the required courses for that program. For more program statistics, see *SI Appendix*, section 3.A.

We consider the same two aggregation strategies across the responses provided by the eight prompting methods: majority vote and maximum performance. For the majority vote, given the eight prompting strategies we have, the final answer to the question is the one that is the most frequent across all strategies. In the maximum performance aggregation, only a single prompting strategy is required to answer correctly for the model to be deemed correct in its response, approximating a pseudo-oracle setting that remains contextually realistic, as a user might be able to distinguish the answer when presented with it, even if they could not find it on their own.

In Table 2, we present the number of courses that would be passed by GPT-4 across the 9 tested degree programs for various course passing thresholds *τ* (i.e., the performance that must be achieved to consider the course passed). Our results show that the general course vulnerability observed in the previous section extends to program vulnerability. At the τ= 50% threshold for passing a course, at least 83% of courses are passed in each of the evaluated programs. In certain programs, such as the Physics Bachelor and Computer Science Master, all tested courses are passed. While this number drops as we raise the passing threshold *τ*, the maximum performance for each program typically remains above 80%, indicating that a combination of clever prompting and partial subject knowledge may be sufficient to achieve high marks on assessment questions.

**Table 2.** Program results

	% Courses passed				Question
Program	*τ* = 50%	*τ* = 60%	*τ* = 70%	Max	count
Engineering	80.0	60.0	40.0	0.83	1,343
Chemistry	83.3	66.7	50.0	0.85	1,417
Life science	85.7	71.4	57.1	0.85	1,477
Physics bachelor	100.0	55.6	33.3	0.86	958
CS bachelor	91.7	66.7	50.0	0.87	1,487
CS master	100.0	83.3	50.0	0.87	1,514
Data science master	90.0	70.0	30.0	0.86	1,576
Physics online	100.0	63.6	27.3	0.84	837
Life science online	85.7	71.4	57.1	0.75	996

For each program, the first three columns show the percentage of courses for which GPT-4 surpasses the thresholds *τ* = 50, 60, 70% correctly answered questions using the majority vote strategy. “Max” represents the proportion of questions in this degree correctly answered by at least one prompting strategy. Program levels are specified as Bachelor, Master, or Online. The first three programs (Engineering, Chemistry, Life Science) are first-year Bachelor course programs.

**Fig. 3.**
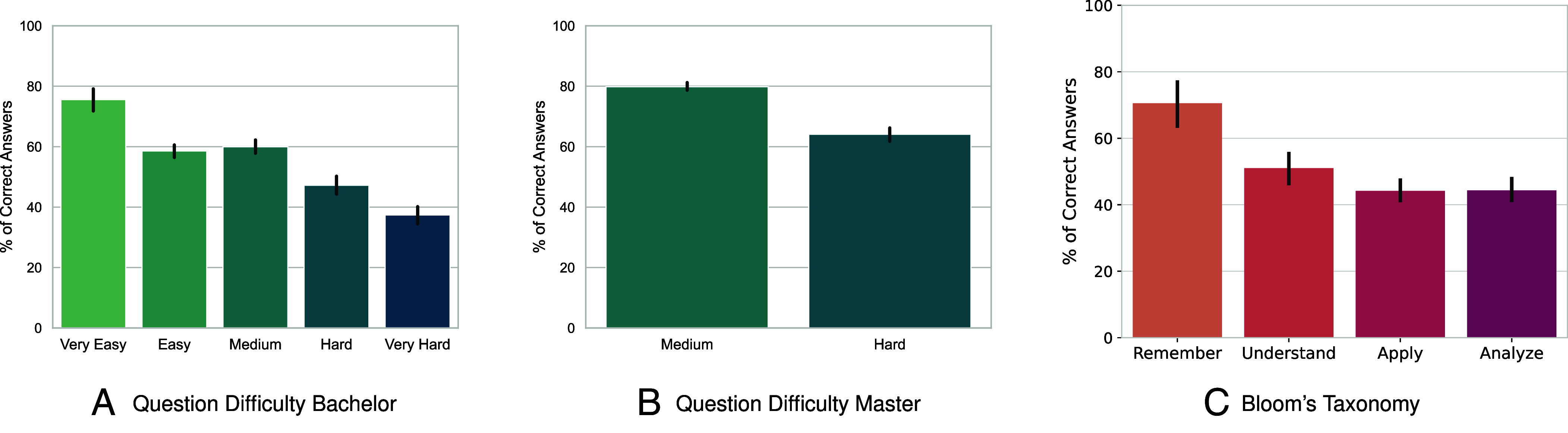
Model Performance Stratified by Question Difficulty. (*A* and *B*) 376 Bachelor’s and 693 Master’s questions, respectively, annotated using instructor-reported difficulty levels. (*C*) 207 questions annotated using Bloom’s taxonomy by two researchers in the learning sciences. Across all categorization schemes, GPT-4 performance slightly degrades as the questions become more complex and challenging. Performance is aggregated by the majority vote strategy. Error bars represent 95% CIs using the nonparametric bootstrap with 1,000 resamples.

Topically, we find that the models consistently exhibit lower performance on assessments of courses involving mathematical derivations. Conversely, the model demonstrates strong performance on problems that have straightforward generation formats, such as text or code. For example, models consistently yield high performance in subjects such as *Software Engineering* and *Intro to Machine Learning*. This observation is further supported by the difference in performance between Master’s and Bachelor’s level courses (shown across Fig. 3 *A* and *B*). In our dataset, Bachelor’s courses feature more mathematical derivations, while Master’s courses have more text and code-based problems. In *SI Appendix*, section 5.A, we provide further performance comparisons between the courses representing each program. In *SI Appendix*, section 5.B, we analyze model performance across all prompting strategies and both question types.

**Fig. 4.**
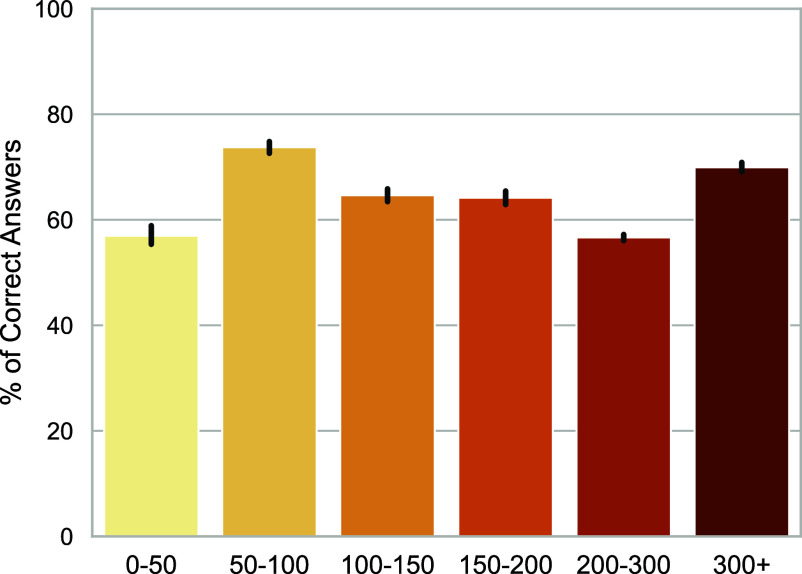
Course Performance by Course Size. Average course performance of GPT-4 with the majority vote strategy stratified by the course size, measured by the number of enrolled students. GPT-4 successfully answers questions for assessments in some of the largest courses by enrollment, amplifying the potential impact of assessment vulnerability. Error bars represent 95% CIs using the nonparametric bootstrap with 1,000 resamples.

Finally, we highlight that these models are effective in courses that large portions of the student body must take, increasing the overall vulnerability of course programs. Fig. 4 demonstrates that some of the largest classes on campus, with upward of 300 students, are also some of the most vulnerable, with GPT-4 achieving (using the majority vote strategy) an average performance of 69.9% in these classes (while hovering around 60% for other class sizes). This result is particularly problematic because larger courses are often limited in terms of the monitoring and mitigation strategies they can implement due to the number of students. While smaller courses may more easily be able to combat the misuse and unethical use of generative AI tools, larger courses, which are often mandatory for degree completion, must ensure fair and scalable solutions for a larger student population.

### More Challenging Assessments Are Only a Half-Solution.

One possible solution to mitigate assessment vulnerability would be to increase their difficulty beyond what generative AI systems are capable of solving, as we observe that the performance of these systems does decrease on more challenging assessment questions (Fig. 3). We measure the difficulty using a subsample of our question bank that is annotated according two different categorizations of their difficulty: 1) *instructor-reported question difficulty*, a five-point difficulty categorization for Bachelor courses and two-point for Master’s courses provided by the course instructors, and 2) *Bloom’s taxonomy* (18), a six-point scale that measures the cognitive complexity of a question.^‡^

For the instructor-reported difficulty categorization, we collect annotations from course instructors for a subset of 376 questions from the Bachelor’s program (n.b., the instructors that designed the questions). The instructor-reported rating ranges from “Very Easy” to “Very Hard” on a 5-point scale. We also collect 693 questions from the Master’s program annotated on a 2-point scale, ranging from “Medium” to “Hard” (the original scale was meant to be 3-point, but no instructor reported an “Easy” question). In Fig. 3 *A* and *B*, we show the model’s performance on questions stratified by their difficulty rating and observe that GPT-4 performs worse on questions that instructors deem harder. For example, in Bachelor courses, there is a 38% difference in accuracy between “Very Easy” and “Very Hard” questions. However, the differences between Bachelor’s “Easy” and “Hard” questions or Master’s “Medium” and “Hard” questions are only 11.5% and 15.75%, respectively.

This pattern is also observed in our assessment of question difficulty performed using Bloom’s taxonomy, which classifies educational learning objectives into levels of complexity and specificity: remember, understand, apply, analyze, evaluate, and create. Two researchers in the learning science manually annotated 207 questions from our dataset according to Bloom’s taxonomy (18). While the taxonomy typically associates questions into six categories, we found that most course assessment questions were covered by the first four categories. The results, grouped by taxonomy category in Fig. 3*C*, illustrate that GPT-4 performance is negatively correlated with the cognitive complexity of the question, with higher performance on lower-level tasks compared to higher-level analysis and application tasks.

**Fig. 5.**
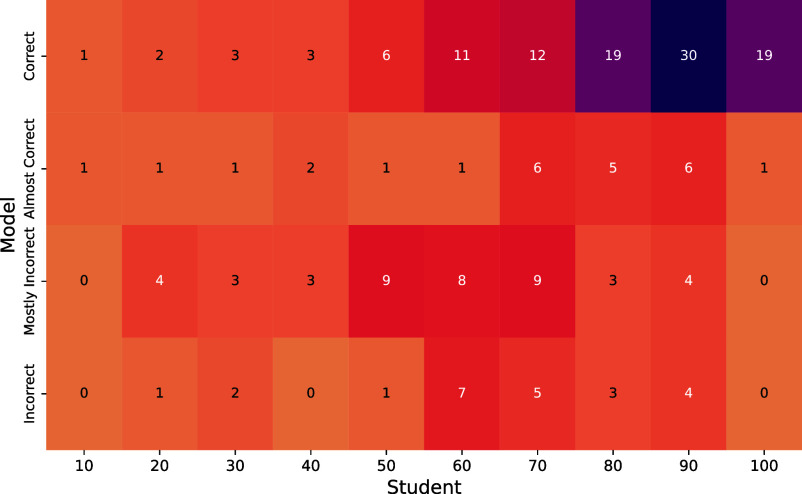
Comparison of Student Performance and GPT-4. Average student performance for a subset of 197 questions is computed and stratified along 10-point intervals from 0 to 100. The model’s performance with the majority vote strategy is assessed by human graders using a 4-point scale. We observe the model typically correctly answers questions that students also answer correctly.

However, harder assessments may not necessarily be a suitable solution for this vulnerability as they would also likely lead to lower student performance. When comparing the performance of students and GPT-4 on problem sets from a subset of questions for which student performance statistics were collected (Fig. 5), we note that the model tends to excel on questions where students also perform well. This pattern perhaps exacerbates fairness as GPT-4 (and similar models) could be used to achieve average results on an assessment while providing few benefits to students who can already complete the easier portion of assessments but struggle with harder questions. Notably, however, we observe that for a subset of problems, the model typically struggles, receiving “Mostly Incorrect” or “Incorrect” marks, while students demonstrate relatively strong performance, with scores ranging from 0.5 to 0.9. These problems typically require mathematical derivations and extensive computations.

## Discussion

### Summary.

In this work, we tested the ability of LLMs to solve assessment questions for a large number of courses from technical and natural sciences academic programs at EPFL. We find that LLMs are generally capable of answering 50 to 70% (depending on the model) of questions correctly given no subject-related knowledge, and up to 85.1% of questions correctly when some subject-specific knowledge is assumed (i.e., the ability to recognize the correct answer). Most importantly, when considering performance across programs, GPT-4 can achieve greater than 50% performance for 83% to 100% of courses (depending on the program) with an average program pass rate of 91.7%. While a higher per-course passing threshold of 70% would only result in 23% to 50% of courses being passed across our programs (with an average of 37%), this would also lead to higher student fail rates as passing thresholds would similarly affect them. Given that continuous advancements in LLM technology will likely further improve these LLM performance numbers in the future, we conclude that higher-education assessment schemes are immediately vulnerable to student exploitation of generative AI tools, specifically in the engineering and natural sciences.

### Assessment Vulnerability.

Our results indicate that the broad availability of generative AI tools should urgently trigger discussion on the design and implementation of assessments. Naturally, our results must be placed in the context where they would normally be observed. In many educational institutions, student assessments are frequently closed-book, thereby precluding the direct use of generative AI tools. Many course assessments (e.g., assignments), though, are completed at home without supervision. In the same vein, most online courses typically evaluate students without any supervised, in-person assessment. In these unsupervised settings, the availability of generative AI tools for aiding in the completion of assessments poses risks for many commonly used student evaluation methods.

One general trend that we observe from our data (Fig. 3*A*–*C*) is that models perform well on basic learning tasks, such as memorizing factual knowledge. In courses where memorization of factual knowledge is a core evaluation objective, students should not be allowed to use these tools in nonproctored settings, and these courses should perhaps return to traditional in-person examination (19). Barring this possibility, in the case of nonproctored assessments, we recommend that their design should not only consider the possibility of assistance from generative AI but actively assume its use. At the very least, assessments should be designed with generative AI in the loop to design AI-adversarial evaluations that remain fair to students.

At the same time, these findings provide an opportunity to improve and diversify student learning through assessments. Students acquire relevant skills when assessments emphasize analytical and applied knowledge settings (20). Rather than using proctored exams, then, or limited practical works, such as assignments, students should be evaluated using assessments requiring a more composite application of course concepts, such as broader class projects. Project settings more closely assess students on problems resembling real-world challenges, would provide students with more opportunities to make problem-solving decisions, such as problem simplification, decomposition, and planning (21), and would mitigate the impact of generative AI tools (Fig. 3*C*).

### Education Vulnerability.

While our results point to an urgent need to mitigate assessment vulnerabilities in higher education, a longer-term view requires considering how education as a practice should evolve alongside the availability of generative AI tools. Since the release of ChatGPT, ongoing discussions have revolved around this topic with both negative and optimistic views. Although numerous studies explore the ways AI can enhance learning, ethical concerns related to plagiarism, biases, and overreliance on technology have also been highlighted (22–28).

An important dimension of these discussions emphasizes the need to revisit evaluation procedures to ensure that students acquire necessary skills and critical thinking abilities in the face of AI adoption (29–32). For instance, observations from various works (and our study) show that models excel in generating code to solve software problems (33–37). While this capability reduces the burden on professional (and hobbyist) software developers, it also poses a risk for learners by potentially offering shortcuts that impede the acquisition of fundamental coding and engineering skills (38). Coding tools such as GitHub’s Copilot or OpenAI’s Codex may lead novices to overrely on autosuggested solutions. This overreliance may diminish their engagement with computational thinking (29, 30), which is arguably the most important skill that is learned in any computer science course or program.

Beyond this example, many studies underscore the significance of adapting course materials and assessments to promote critical thinking, encourage student collaboration, develop practical skills, enhance soft skills, and promote interdisciplinary learning, all with the aim of cultivating graduates equipped with a diverse range of competencies (32, 39–41). In particular, reinforcing our conclusions above, open-ended assessments are proposed to promote originality and creativity, potentially discouraging reliance on generative AI tools and fostering unique ideas and critical analysis (41, 42). One example of program reconsideration is teaching students at computer science courses prompt engineering, which would be essentially programming in natural language (43). This would prioritize problem-solving and higher-level concepts over the technical syntax of programming languages. Ultimately, many of these studies suggest the greater risk of generative AI may be its potential to enable the unintentional circumvention of the frameworks by which learners are taught the foundations to learn later skills, and that teaching and assessment should be adapted for this risk.

Finally, assuming that students will use and become acquainted with the capabilities of these technologies, we recommend that students should not only be educated on the technical and ethical challenges of generative AI systems but also on the critical thinking required to successfully engage with such tools (44). One such measure could involve establishing committees for ethical oversight and adding classroom discussions on the use of AI tools. Such discussions would clarify what constitutes plagiarism and address potential ethical concerns, ensuring that students understand the importance of academic integrity and discern the boundaries between legitimate assistance and academic misconduct (31, 38–42).

## Limitations

While our study offers preliminary insights into the vulnerability of degree programs to student use of AI assistants for assessments, we acknowledge several limitations in our study.

First, our study excluded any multimodal inputs, such as questions containing diagrams, figures, or graphs, which were omitted from our dataset. Approximately 57% of the initially collected data did not qualify for inclusion in the final dataset of 5,579 questions. Consequently, models were solely evaluated with text-only questions. This approach likely resulted in performance outcomes that are higher than what these models would attain when tested on question sets that include these other modalities, though we also note rapid growth in the multimodal capabilities of these models (45).

Our results for GPT-4’s performance on open-answer questions may have also been slightly overestimated because we also used GPT-4 model as a grader As this dual use of GPT-4 could introduce potential grading bias (46), we compared the grades provided by GPT-4 to human scores on a subset of the questions. When comparing the alignment between human-assigned and model-assigned grades for responses from both GPT-4 and GPT-3.5, our results show minimal bias toward GPT-4’s responses relative to GPT-3.5.

Simultaneously, our findings might underestimate the performance potential that students could attain through collaboration with these systems. Although we conducted a thorough examination of prompting strategies, our methods are limited by the fact that they 1) rely solely on published prompting strategies, 2) are generally noninteractive, and 3) are tailored for scalability across all questions to facilitate a comprehensive study. Students aiming to address individual questions could devote more time and devise more interactive, less standardized prompting strategies to collaboratively guide the models toward improved solutions.

Finally, we acknowledge certain gaps between our evaluation of AI systems in this study, and how students are normally evaluated in these courses. First, our study standardizes system evaluation across all course assessments, removing course-specific assessment policies for questions. For example, certain courses, beyond not giving points for correct answers to multiple-choice questions, might also penalize incorrect answers more than leaving a question unanswered, while our study simply gives zero points for incorrect answers. Second, our dataset of questions for each course is not necessarily balanced according to a course’s grading rubric. As an example, our dataset may contain a balanced mixture of questions from assignments and exams for a particular course, while the overall evaluation of a student in this same course would compute their grade as a 10% mixture of assignments, and 90% mixture of exam questions. Similarly, many courses at our institution also include lab or project components as part of their final grade. Since these parts of the assessment do not have a “correct answer” that can be easily marked, they are not included in our dataset.

As we do not consider these course-specific assessment policies when computing the course pass rates of our tested AI assistants, these design decisions introduce a gap between our evaluation and the actual assessment rubrics by which students are graded in our institution’s courses. Despite this divergence, however, we note that other institutions may implement course assessments and grading rubrics in different ways. As a result, while our study is not an exact simulation of our institution’s diverse assessment schemes among its courses, it remains a suitable testbed for providing insights into how course assessments are vulnerable to AI assistants, and how this vulnerability would extend to full university programs without mitigations.

## Materials and Methods

In this section, we provide further details on our data collection process, the prompting strategies used for response generation, and our pipeline for automated grading.

### Dataset Collection.

Our data collection was approved by the Human Research Ethics Committee at EPFL. Data were voluntarily submitted by members of the Data Consortium, and no materials were used without the permission of the data owner.

### Dataset Preprocessing.

To preprocess our data, we collect assessments from participating faculty, extract questions and answers from these assessments, and standardize them into a uniform format. After compiling an initial question bank from the raw data, we filter unsuitable data points by 1) removing questions that lack the question body or solution, 2) eliminating duplicate questions, and 3) removing questions that require information that cannot be parsed by LLMs in a textual format (e.g., diagrams, images, plots). In cases where a joint context is provided for multiple questions, we augment each question individually with this context.

### Prompting Strategies.

To generate answers to questions, we employ various prompting strategies requiring only familiarity with relevant literature and minimal adaptation. We selected eight distinct prompting strategies that we broadly categorize into three types: direct, rationalized, and reflective prompting. Under direct prompting, we use zero-shot, one-shot (9), and expert prompting (10), where models are directly prompted for an answer without encouraging any underlying rationale. For rationalized prompting, three strategies are implemented: zero-shot (12) and four-shot chain-of-thought (11), and tree-of-thought (13) prompting. Here, language models are prompted to generate a rationale before providing the final answer. Last, reflective prompting includes self-critique (14, 15) and metacognitive prompting (16), where models are asked to reflect on a previously provided answer and adjust their response according to this reflection. In our experiments, we noted that the prompting strategy substantially influences model performance, with at least one strategy consistently producing the correct answer in the majority of cases. A detailed description of all prompting strategies, along with prompts, is provided in *SI Appendix*, section 2.

### Evaluation.

In this section, we outline the grading strategies used to evaluate the model’s performance across two question types: multiple-choice (MCQ) and open-answer questions. For MCQ, grading is automated by comparing against the annotated answer. Answers receive a binary score of 0/1 if only one correct option exists, or a proportional score based on the number of correct choices made for multianswer questions (with no penalty for wrong choices). *SI Appendix*, section 4.A provides more details for grading MCQs. For open-answer questions, we constructed a multistep evaluation pipeline using GPT-4 as a grader (7), which we describe below. For both types of results, we report error bars representing 95% CIs (Figs. 3 and 4). These intervals were computed using the nonparametric bootstrap with 1,000 resamples. We also tasked human experts with independently grading a subset of model responses to measure alignment between model and human grading and establish a confidence level for model-based grading.

#### Automated grading.

A significant portion of the questions we extracted are open-answer questions, which are challenging to evaluate manually due to the sheer volume of responses (a total of 33,904 answers from 2,119 questions, answered by 2 models using 8 prompting strategies). As a result, we use a state-of-the-art LLM, GPT-4, as a grader. To automate the grading of open answers, we provide the model with the question, the correct solution from an answer sheet of the assessment, and the generated answer text, prompting it to assign a rating based on the quality of the response. We provide the model with a 4-point grading scale: Correct, Mostly Correct, Mostly Incorrect, Incorrect. The model is first tasked with assessing the accuracy and completeness of the answer before assigning the final grade. Although we do not use these interim accuracy and completeness scores, we manually observe that these stages enhance the quality of overall grading. The specific prompting strategy is detailed in *SI Appendix*, section 4.B. As an answer was produced for each question using eight distinct prompting strategies, we obtained eight different answers and corresponding grades. To present a cohesive performance score for both GPT-4 and GPT-3.5 for a given question, we employ two aggregation methods: 1) the *maximum* approach, which selects the answer with the highest grade for each question as a representation of model performance, and 2) the *majority* approach, which considers the grade that appears most frequently among the eight prompting strategies. As an example, for a hypothetical question whose generated answers for the eight prompting strategies received 2 Correct, 2 Mostly Correct and 4 Mostly Incorrect grades, the *maximum* grade would be Correct and the *majority* grade would be Mostly Incorrect. To report dataset-level performance, we map grade ratings for each example to a score from a discrete range between 0 and 1: {Correct: 1.0, Mostly Correct: 0.66, Mostly Incorrect: 0.33, Incorrect: 0.0} and average the scores.

#### Human grading.

To assess how well model grading aligned with human grading on open-answer questions, we enlisted 28 expert annotators from the teaching faculty of 11 courses to evaluate 933 questions. The courses chosen for human expert grading are listed in *SI Appendix*, section 4.C. Specifically, we requested graders to assign scores to open-ended responses generated by GPT-4 and GPT-3.5. Responses for human grading for both models were generated using two prompting strategies: zero-shot chain-of-thought prompting (11) (a simple prompting method at the disposal of any student) and metacognitive prompting (16) (one of the most effective strategies across all courses). We anonymized the outputs to prevent graders from knowing which model and prompting strategy they were evaluating. To maintain consistency, we instructed graders to use the same grading scale as GPT-4’s direct grading. The number of graders per course varied from 1 to 10, and a total of 3,732 answers were evaluated. On average, graders spent approximately 5 min assessing each answer.

**Fig. 6.**
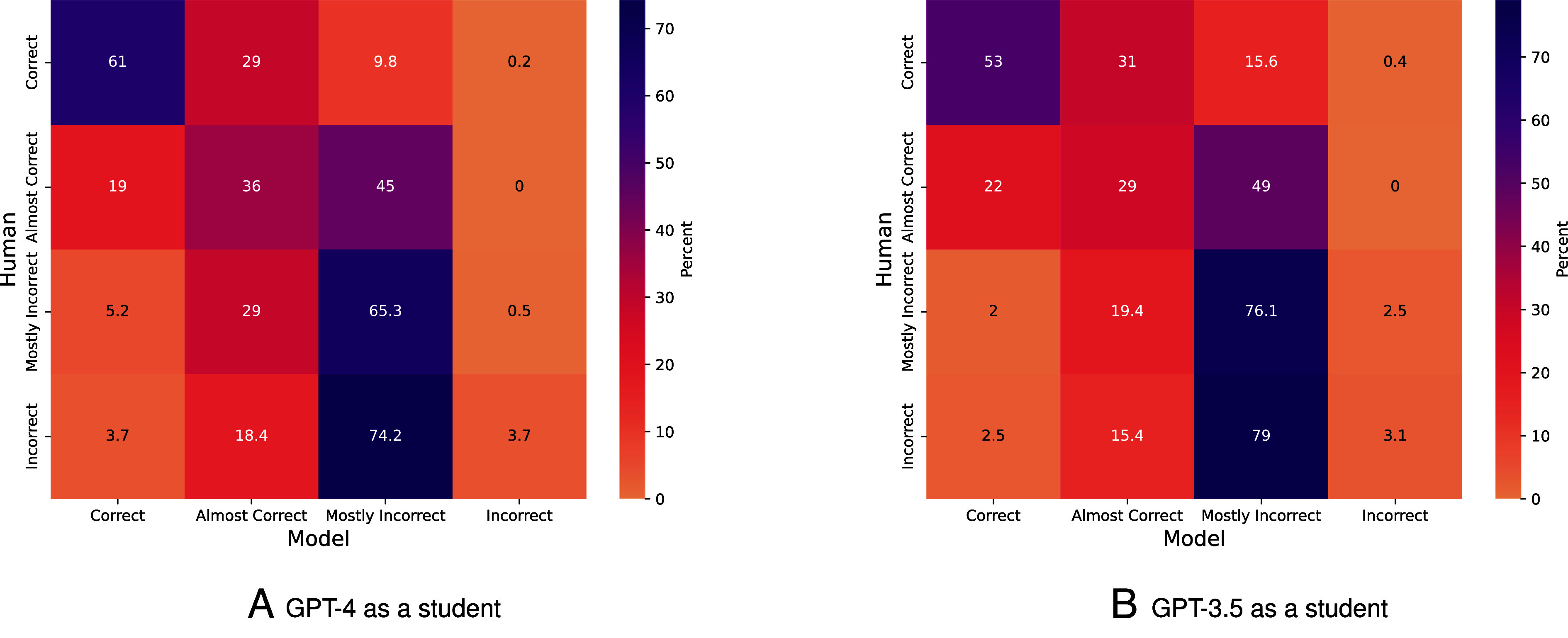
Comparison of Human and GPT-4 grading. Average model and human performance for a subset of 933 questions and answers from (*A*) GPT-4 and (*B*) GPT-3.5 generated with the metacognitive prompting method.

Fig. 6 indicates a general alignment between human graders and GPT-4 when categorizing answers into a simplified correct/incorrect quadrant. Out of the examples identified as Correct by graders, the model assigned the same grade to 61% of them. Similarly, for examples graded as Almost Correct by graders, the model’s grade matched in 36% of cases. Additionally, in instances where graders labeled examples as Mostly Incorrect, the model’s grade aligned with the grader’s assessment 65% of the time. However, we note certain patterns of discrepancy. For instance, GPT-4 as a grader tends to avoid explicitly labeling solutions as Incorrect, and instead opts for Mostly Incorrect (i.e., in 74% of cases that humans annotated a solution as Incorrect, the model identified it as Mostly Incorrect), potentially due to the practice of aligning models for harmlessness (47). We find a few instances where the model rates an answer as Correct while humans assign a lower score.

Interestingly, upon comparing average grades assigned by human graders and GPT-4 across 11 courses, we find a difference in average grade of only 2.75%. However, we observe variations between courses, with an average course grade deviation of 8.5% (and the largest deviation for a course being 26%) between human and model graders. Finally, we also note the performance correlation between MCQ and open-answer questions in Fig. 2, providing a comparison point for the rationality of our model-based open-answer grading results. While scores for open-answer questions are typically lower than MCQ, the patterns exhibited by both curves are similar across both models. Overall, we note that the grades provided by humans and models are moderately correlated and that the summary statistics across courses tend to have a high correlation. Additional results, such as further comparison of human and model grades for additional prompting strategies, pairwise agreement scores between human and model graders, and qualitative human assessments of the responses for both GPT-3.5 and GPT4, can be found in *SI Appendix*, section 4.

#### Automated grading in prior work.

A substantial body of research leverages LLMs for response evaluation. Traditionally, automated assessment has necessitated high-quality reference data obtained through human grading, which is both costly and time-intensive. Consequently, there has been considerable exploration into the potential of LLMs to serve as evaluators (48). Recent research has found LLMs to be capable of generating quality feedback (15, 49–55), a trend also reflected in investigations into LLM-based evaluation (7, 56–59).

Automated solutions for student grading have been explored in the field of learning science, as well, some of which now use LLMs (60). Intelligent Tutoring Systems, such ALEKS (61), ASSISTments (62), Cognitive Tutor (63), and MATHia (64) are widely employed to automatically assess student performance in closed-ended questioning. These systems cater to several hundred thousand students annually (62, 65). Meanwhile, AES platforms such as e-Rater (66), IntelliMetric (67), and Intelligent Essay Assessor (68) have emerged as useful tools for evaluating numerous student essays and responses to open-ended questions each year (67–71).

## Supplementary Material

Appendix 01 (PDF)

## Data Availability

The data and code are available under an open-source license to facilitate further research and collaboration within the community. The course data, model responses, and code can be accessed at the GitHub repository (https://github.com/epfl-nlp/nlp4education) (72). The dataset has been anonymized to ensure compliance with privacy regulations.

## Footnotes

^*^As of November 2023.

^†^Analysis of the quality of this automated grading is provided in *Materials and Methods* and *SI Appendix*, section 4. Importantly, we note that GPT-4 gives slightly higher average grades (on average ∼2.75%) than humans for responses to a sample of questions graded by both.

^‡^More details about Bloom’s Taxonomy can be found in *SI Appendix*, section 3.B.
