# Supplementary material for: Could ChatGPT get an engineering degree? Evaluating higher education vulnerability to AI assistants
Source: Proc Natl Acad Sci U S A. 2024 Nov 26;121(49):e2414955121. doi: 10.1073/pnas.2414955121 (PMC11626143; doi:10.1073/pnas.2414955121)
Supplement: Supplementary file 1 — Appendix 01 (PDF) [file pnas.2414955121.sapp.pdf]

Supporting Information for

Could ChatGPT get an Engineering Degree? Evaluating Higher Education Vulnerability to AI Assistants

Beatriz Borges[1], Negar Foroutan[1], Deniz Bayazit[1], Anna Sotnikova[1], Antoine Bosselut

Corresponding author: Antoine Bosselut. E-mail: antoine.bosselut@epfl.ch

This PDF file includes:

Supporting text
Figs. S1 to S2
Tables S1 to S11
SI References

Supporting Information Text

1. OpenAI API Hyperparameters

For both GPT-4 and GPT-3.5, we set `temperature=0.8` to increase the diversity and encourage more creative responses. Moreover, we set `presence_penalty=0.5` and `frequency_penalty=0.8` to reduce repetitive samples while keeping the quality of the generations high. These values are chosen based on a human evaluation of the fluency and quality of the responses given a set of questions. For the rest of the hyperparameters, we use their default values.

2. Prompting Strategies

Our study's goal is to identify the vulnerability of educational assessments to AI systems. As a result, we select prompting strategies that simulate realistic student use and assess prompting strategies that can be used with minimum effort, requiring only knowledge of the relevant literature and minimal adaptation. We exclude strategies involving training models. Our assessment encompasses three primary categories of prompting strategies: *direct prompting*, wherein the model is directly prompted to provide an answer; *rationalized prompting*, which encourages the model to first verbalize reasoning steps before providing a response; and *reflective prompting*, which prompts the model to reflect on a previously generated response before finalizing an answer. Each prompt is tailored for three scenarios: (1) MCQs with a single correct answer, (2) MCQs with multiple correct answers, and (3) open-answer questions. Below, we outline the strategies used to prompt models to answer questions:

A. Direct Prompting. We explore three strategies for direct prompting: zero-shot, one-shot, and expert prompting. All these strategies ask the LLM directly for an answer without encouraging any particular strategy or rationale to arrive at the answer.

Zero-shot Prompting. We ask the model to solve questions without any demonstrations or system role prompts. The instructions vary depending on the type of question: open-answer or multiple-choice (MCQ). Additionally, we differentiate between multiple-choice cases where a single answer is correct and cases where multiple correct answers can be selected.

> **MCQ single answer**: You are given a question followed by the possible answers. Only one answer is correct. Output the correct answer.
>
> **MCQ multi answer**: You are given a question followed by the possible answers. The question can have multiple correct choices. Output all the correct answers.
>
> **Open answer**: Solve the following question:

For MCQs, each answer option is associated with a letter, and the model is expected to provide the letter corresponding to its choice.

One-shot Prompting (1). In this prompting strategy, we instruct the model to solve questions based on a provided example as a demonstration, without any additional system role prompt. Each question is paired with a demonstration that is the most similar to the question being addressed. Specifically, we use the "all-roberta-large-v1" model* (3) to embed all questions as vectors, and then retrieve the most similar question vector based on cosine similarity to the prompt question vector. We append the corresponding demonstrative example to this retrieved vector to the prompt. The prompt instructions remain the same as for zero-shot prompting. The demonstration is provided to the model in a multi-message setting, mimicking an actual conversation between the user and the assistant.

Expert Prompting (4). In expert prompting, we use the LLM to to simulate the responses of three experts in the field. The model generates answers as if written by these experts, and then we combine their responses using collaborative decision-making, typically through majority voting. This process is represented by using a generic expert defined as the system role, such as "*You are a professor of Machine Learning*" for questions from, e.g., Machine Learning† and prompting the model to give us the names of three experts in the field capable of solving the given question using the prompt:

> **System:** You are an expert in {*course name*}.
>
> Give an educated guess of the three experts most capable of solving the following question. Only output the name of these three experts as a json format with key as number and value as a name, without any explanation.

Following this, the model adopts the personas of the named experts as its system role to produce an answer, employing the same prompt as used in the zero-shot and one-shot strategies. The answers generated by these personas are then aggregated using a majority voting approach.

B. Rationalized Prompting. We explore three strategies for eliciting reasoned answers: zero-shot and four-shot chain-of-thought, and tree-of-thought prompting. Each strategy involves prompting the LLM to generate a rationale before providing a final answer.

*We use the sentence-transformers (2) implementation of this model, available at https://huggingface.co/sentence-transformers/all-roberta-large-v1.

†Whenever a prompting strategy makes use of a course name, it is employing the course name rather than the course code (e.g., "Machine learning for physicists" rather than course code like "PHYS 444").

 Beatriz Borges[1], Negar Foroutan[1], Deniz Bayazit[1], Anna Sotnikova[1], Antoine Bosselut

Chain-of-Thought Prompting (CoT) (5). In chain-of-thought prompting, we guide the model to generate a sequence of reasoning steps before providing an answer. This approach typically results in more coherent, structured, and accurate responses, as it requires the model to present arguments before delivering the final answer. This behavior is often initiated by an instruction such as "Let's think step by step." For better performance, the model may be given demonstrations that illustrate how to break down a question into multiple reasoning steps. However, manually generating these demonstrations for each course is time-consuming. Therefore, we automatically generate multiple example rationales using GPT-4 for questions from each course. Domain experts then manually select the best chain-of-thought reasoning trace for each question and correct or improve it if necessary.

We experiment with two settings: zero-shot (6) (no demonstration) and few-shot (4 demonstrations). For the latter, for each question, we sample 4 demonstrations of the same course cluster (same topic) and the same question type (MCQ or open-answer), ensuring that these demonstrations were different from the question being asked to the model. Sometimes, the total length of the 4 demonstrations exceeds the model's maximum context length. In such cases, we reduced the number of demonstrations to fit within the context limit. Additionally, we provided a system prompt that included the course topic as an extra hint for the model. For each question type, the selected prompts are the following:

> **System:** You are an expert in {*course name*}.
>
> **MCQ single answer**: You are given a question followed by the possible answers. Only one answer is correct. Give a step-by-step reasoning, and then output the correct answer.
>
> **MCQ multi answer**: You are given a question followed by the possible answers. The question can have multiple correct choices. Give a step-by-step reasoning, and then output all the correct answers.
>
> **Open answer**: Solve the following question, by first giving the step-by-step reasoning and then outputting the answer:

The demonstration pairs, which include the question and its reasoning explanation, are provided to the OpenAI API using a multi-message setting similar to the few-shot strategy.

Tree-of-Thought Prompting (7). While chain-of-thought prompting has led to performance improvements in many NLP tasks, it is sensitive to incorrect reasoning steps, as there is no mechanism to assess and fix a reasoning error after it has been made. Tree-of-thought prompting extends chain-of-thought by having the model emulate three subject experts. Each of them must generate a reasoning path and critique the other expert's proposed paths. Then, the model is instructed to simulate a discussion between experts until they reach an agreement and provide a final answer. We use the following prompt to implement Tree-of-Thought:

> **System:** You are an expert in {*course name*}.
>
> Imagine three different experts answering this {*question type*} question. They will brainstorm the answer step by step reasoning carefully and taking all facts into consideration.
> All experts will write down one step of their thinking and then share it with the group. They will each critique their response and all the responses of others. They will check their answer based on science. Then all experts will go on to the next step and write down this step of their thinking. They will keep going through steps until they reach their conclusion taking into account the thoughts of the other experts. If at any time they realize that there is a flaw in their logic, they will backtrack to where that flaw occurred. If any expert realizes they're wrong at any point then they acknowledge this and start another tree of thought. Each expert will assign a likelihood of their current assertion being correct. Continue until the experts agree on the single most likely answer.

C. Reflective Prompting. We explore two strategies for reflective prompting: self-critique and metacognitive prompting. Both strategies involve the model reflecting on an answer it previously provided. Based on this reflection, the model then generates a final, improved answer.

Self-Reflect Prompting (8, 9). This strategy is performed on in conjunction to CoT to refine the reasoning traces generated by the model. Focusing on MCQ questions, first, we provide the model with a question and its zero-shot CoT response. Then, we prompt the model to revise its reasoning and produce a refined answer. Notably, this refinement process is carried out without any demonstrations.

> {*CoT prompt and model output*}
>
> **MCQ single answer**: Please consider that there is a single correct choice. Is the provided reasoning accurate? If there isn't any inaccuracy, please output "Reasoning is fine." Otherwise, please revise your reasoning and then choose the single correct choice.
>
> **MCQ multi answer**: Please consider that multiple choices can be correct. Is the provided reasoning accurate? If there isn't any inaccuracy, please output "Reasoning is fine." Otherwise, please revise your reasoning and then and then output all the correct choices.
>
> **Open answer**: Assume you got the above answer from a student and you're looking for inaccuracies in either the reasoning or the final response. Try to refine any inaccuracy and answer the question from scratch. Please don't mention in your answer that you're refining a previous answer and write a new answer from scratch. Answer:

Metacognitive Prompting (10). Motivated by the concept of meta-cognition, this prompt is designed to emulate the human process of introspection and regulation of thinking. To achieve this, the language model is tasked with following a specific procedure akin to human cognitive processes. This involves sequentially: (1) deeply understanding the problem, akin to human comprehension; (2) identifying relevant concepts and formulating a preliminary answer; (3) evaluating and adjusting this preliminary answer if needed; and (4) confirming the final response and presenting it in a specified format.

You have to answer the following {*question type*} question.
{*Question text*}
As you perform this task, follow these steps:
1. Clarify your understanding of the question.
2. Make a preliminary identification of relevant concepts and strategies necessary to answer this question, and propose an answer.
3. Critically assess your preliminary analysis. If you are unsure about its correctness, try to reassess the problem.
4. Confirm your final answer and explain the reasoning behind your choice.

3. Dataset Details

A. Program Statistics. In our work, we study nine programs from three program levels: Bachelor, Master, and Online. Table S1 shows the number of courses available per program.

Table S1. Program Statistics. "Required" shows the ratio of required courses present in our data over the total number of required courses per program. "Optional" shows the number of optional courses per program. "Total" shows their sum, that is, the total number of courses our dataset covers, per program.

Program	Number of Courses		
	Required	Optional	Total
Engineering, 1st year BSc	5/12	-	5
Chemistry, 1st year BSc	6/9	-	6
Life Science, 1st year BSc	7/11	-	7
Physics, BSc	8/26	1	9
Computer Science, BSc	10/21	2	12
Computer Science, MSc	5/10	3	8
Data Science, MSc	3/9	7	10
Physics, Online	-	-	11
Life Sciences, Online	-	-	8

B. Bloom's Taxonomy. Bloom's taxonomy (11) is a framework for categorizing learning objectives and educational items into levels of complexity requiring different cognitive skills. The taxonomy consists of 6 levels, from basic knowledge recall to higher-order critical thinking. The lower levels (*remember*, *understand*, and *apply*) focus on foundational cognitive tasks such as remembering facts and comprehending basic information. As learners progress to higher-level categories, they engage in more complex cognitive tasks. The upper levels (*analyze*, *evaluate*, and *create*) emphasize critical thinking, problem-solving, and creativity.

Although Bloom's Taxonomy is widely accepted and used, educators often disagree about the precise definitions of each category. This discrepancy leads to varied interpretations and challenges in categorizing learning objectives and educational items into specific taxonomy levels. This is particularly true when moving between adjacent levels, such as *understand* and *apply*. For example, given the following MCQ:

```
In which of the following cases does JOS acquire the big kernel lock?
Options:
A. Processor traps in user mode
B. Processor traps in kernel mode
C. Switching from kernel mode to user mode
D. Initialization of application processor
```

On the one hand, to solve this question correctly, it is required to recall specific knowledge (*remember*) about the circumstances under which JOS acquires the big kernel lock from the lecture or other learning materials. However, it can also be classified as the *understand* category, as some multiple-choice options act as distractors that test the depth of a student's comprehension of the topic. As a result, the taxonomy has limitations in addressing the complexities of modern learning environments, especially in blended learning where information access and processing diverge from the conventional classroom setting for which Bloom's Taxonomy was crafted.

Despite these ambiguities, Bloom's taxonomy remains a leading categorization scheme of cognitive difficulty in education. In this work, we assign Bloom's taxonomy labels to various questions in our dataset to assess model performance across questions of varying cognitive difficulty. To assign Bloom's meta-labels to questions in our dataset, we tasked two learning sciences experts to label 207 randomly-selected English questions with one of the six Bloom categories. They achieved an inter-annotator agreement of 51% on this task. Using a more forgiving *fuzzy agreement* (which also indicates an agreement if the annotators select adjacent categories) yields an agreement score of 84%. Results for performance stratified by Bloom's taxonomy label can be found in the main article.

4. Evaluation

In this section we describe the methods used for grading MCQs and open answer questions with GPT-4.

A. Multiple Choice Scoring. Regardless of the prompting strategy used for MCQs, the model is provided with the list of answer choices, each associated with a letter, and is asked to generate the letter(s) corresponding to the correct answer(s). Therefore, grading MCQs involves extracting the letter(s) indicated in the model's response and comparing them with the correct answer(s) (i.e., ground truth).

This process is straightforward for direct prompting strategies, but more challenging for strategies involving reasoning, such as chain-of-thought, where the model's response may include long explanations that discuss incorrect answers. To ensure consistency in answer extraction across different types of responses, we use an GPT-3.5 with the following prompt to extract the model's final answer:

> *{Question prompt}*
> *{Model output}*
> If the above answer does not provide an option or gives an answer which is not in the options list, you should give the following:
> {"selection": [None]}
> Otherwise, please return the answer in a dictionary format, with the key being "selection", and the value is a list that contains the index of letters of all the correct choices, with A being 0, B being 1, and so on:

B. Open Answer Direct Grading. For open-answer grading, we compare the performance of GPT-4 as a grader against human graders from the teaching staff of the courses from which the questions originated.

Grading Open Answer Questions. To automatically grade the quality of open answers, the GPT-4 grader model is given the question, the gold solution (extracted from the course materials), and the text of the generated answer, and prompted to assign a rating to the generated answer based on its quality. Rather than asking the model for a single *correct* or *incorrect* label, we provide the model with a 4-point grading scale, ranging from *correct*, *almost correct*, *mostly incorrect*, to *wrong answer*. The full prompt is presented below:

> **System prompt**: You are a teacher of *{course name}*. You must grade exam questions.
>
> **User Prompt**: You must rigorously grade an exam question. Please be strict and precise in your assessment, providing reasoning for your assigned grade. Here's the process I'd like you to follow:
> Carefully read and understand the question.
> Thoroughly compare the student's answer with the correct golden answer.
> Evaluate the student's response based on its accuracy and completeness.
> Deduce a final grade by considering whether the answer is "wrong answer", "mostly incorrect", "almost correct", "correct", along with a clear explanation for your decision.
> Question: *{question}*
> Gold Answer: *{gold answer}*
> Student Answer: *{model output}*
> format your answer in the following json format, providing a clear and detailed evaluation for each of the two criteria (accuracy and completeness) and finally providing the grade. in the field of grade only write the final grade from the given grading options:
> {"accuracy": ,
> "completeness": ,
> "grade":
> }

C. Comparing GPT-4 and Human Grading. To better understand GPT4's capabilities as a grader, we compare its grading performance against the human grading scores, using two metrics: *Average Grade* and *Grade Agreement*. We recruited 28 graders from 11 of the courses in our dataset and tasked them with providing a general assessment of the quality of 933 responses provided by GPT-3.5 and GPT-4. Similar to GPT-4 as a grader, human graders are asked to use the same 4-point scale to grade model outputs. Given the cost of performing this annotation, we only task graders to mark responses from two prompting strategies, Zero-shot CoT (5) and Metacognitive prompting (10).

Average Grade. To evaluate the similarity between grades given by humans and GPT-4 for each course, we first compare the average grades they provide to responses to questions in each course of our dataset. To quantify the grades given by the model and humans, we map grade ratings to a discrete range between 0 and 1: {*correct*: 1.0, *almost correct*: 0.66, *mostly incorrect*: 0.33, *wrong answer*: 0.0}.

Table S2 shows the average grades provided by both human graders and GPT-4 to question responses generated by both GPT-4 and GPT-3.5. Two prompting strategies were used: zero-shot CoT and metacognitive prompting. On average, for most of the courses, the model tends to give higher grades compared to human graders, particularly for the zero-shot CoT prompting strategy. Some courses show a significant disparity, with GPT-4 giving much higher grades than humans (e.g., *Advanced Computer Architecture* and *Software Engineering*). More rarely, the human graders consistently give higher scores for courses such as *Machine Learning for Physicists* or, to a lesser extent, *Applied Data Analysis*. We also observe variations between the two prompting strategies: for *Mathematics of Data*, humans give higher grades than the model for metacognitive prompting, while the model gives the highest grades for zero-shot CoT. Overall, both GPT-4 and human graders tend to give higher grades to GPT-4 answers than GPT-3.5. Despite these differences, these results also indicate that humans and GPT-4 have a similar grading distribution for model responses (particularly for responses to the metacognitive prompting strategy).

Table S2. Comparison between human graders and the GPT-4 model across multiple university courses. The average grades provided by human graders and the GPT-4 model for open-answer questions. Results are presented for two prompting strategies (Zero-Shot CoT and Metacognitive) and each student model (GPT-3.5 and GPT-4). Each performance is reported with a 95% confidence interval.

Prompting Strategy:	Zero-Shot CoT				Metacognitive Prompting			
**Course Name** Model:	GPT-4 Responses		GPT-3.5 Responses		GPT-4 Responses		GPT-3.5 Responses	
Grader:	Human	GPT-4	Human	GPT-4	Human	GPT-4	Human	GPT-4
Statistical Physics	48.9 ± 10.1	53.7 ± 6.9	43.9 ± 11.4	38.6 ± 4.4	36.4 ± 10.1	45.5 ± 6.3	37.6 ± 10.6	39.9 ± 4.4
Concurrency & Parallel Processing	62.3 ± 14.5	68.4 ± 9.5	62.3 ± 14.5	61.0 ± 8.1	72.8 ± 14.5	68.4 ± 10.5	56.1 ± 15.6	53.8 ± 9.4
Advanced Computer Architecture	50.4 ± 10.5	74.9 ± 6.2	44.9 ± 11.1	68.8 ± 6.9	60.3 ± 11.0	73.1 ± 6.2	42.3 ± 10.5	62.6 ± 6.2
Software Engineering	62.2 ± 9.0	85.9 ± 4.8	47.9 ± 9.5	72.9 ± 5.3	66.1 ± 9.9	84.1 ± 5.8	49.8 ± 10.5	73.0 ± 5.7
Mathematics of Data	52.1 ± 13.5	65.4 ± 8.1	50.2 ± 13.5	56.4 ± 8.1	94.5 ± 5.5	68.9 ± 7.3	76.5 ± 12.7	56.4 ± 9.0
ML for Physicists	80.8 ± 7.2	76.7 ± 5.2	71.7 ± 7.9	69.1 ± 6.3	80.4 ± 6.8	73.9 ± 4.8	74.4 ± 7.6	69.5 ± 5.2
Semiconductor Properties	74.1 ± 15.3	78.6 ± 10.7	66.4 ± 12.2	78.5 ± 12.2	63.4 ± 16.6	66.3 ± 10.6	55.8 ± 15.2	64.8 ± 10.7
Applied Data Analysis	74.8 ± 13.1	72.3 ± 10.8	58.1 ± 13.1	57.9 ± 8.3	73.6 ± 13.1	65.1 ± 9.5	65.2 ± 13.1	55.6 ± 9.6
Advanced General Chemistry	78.9 ± 8.4	80.7 ± 6.6	58.8 ± 10.8	64.0 ± 7.8	80.8 ± 7.8	81.4 ± 7.8	62.3 ± 10.1	62.3 ± 7.8
Information & Communication	76.6 ± 4.7	74.3 ± 3.9	57.6 ± 5.1	56.2 ± 3.9	74.3 ± 4.1	70.9 ± 3.9	60.3 ± 5.1	59.5 ± 3.9
Analysis I	37.2 ± 4.7	48.7 ± 3.2	28.8 ± 4.1	38.4 ± 2.6	48.6 ± 4.1	47.1 ± 3.1	42.9 ± 4.3	44.5 ± 2.8
Average	63.5 ± 9.1	70.9 ± 6.9	57.7 ± 10.3	60.2 ± 6.7	68.3 ± 9.4	67.7 ± 6.9	56.7 ± 10.5	58.4 ± 6.8

Agreement. While investigating average grades provides an initial assessment of whether GPT-4's grading distribution generally matches that of humans, it does not give us a comprehensive understanding of the alignment between GPT-4 and the teaching staff's grading, so we now investigate the level of response-level grade agreement between the two. The agreement is defined as the percentage of question responses for which the model and human give the same grade. Table S3 shows the average rate of grade agreement for each course. For each course, student model, and prompting method, we report the exact agreement between human graders and GPT-4 as a grader. The agreement between the model and the human varies for different courses, changing from $\sim$18% to $\sim$70%, while the average agreement across all courses stays below 50% for both metacognitive and zero-shot CoT. Figure S1 shows the human and GPT-4 assigned grades distribution for both GPT-4 and GPT-3.5 as the student, including the two prompting strategies. We observe that GPT-4 as a grader tends to grade model outputs using the labels *almost correct* and *mostly incorrect* far more often than human graders, while rarely identifying a response as *wrong answer*. In contrast, human graders are more generous at identifying responses as *correct*, but almost more willing to identify responses as *wrong answer*.

Table S3. Pairwise agreement (%) between grades provided by human graders and the GPT-4 model as a grader.

**Course Name**	Pairwise Agreement (%)			
	Zero-Shot CoT		Metacognitive	
	GPT-4	GPT-3.5	GPT-4	GPT-3.5
Statistical Physics of Computation	26.4	20.8	22.6	18.9
Concurrency and Parallel Processing	37.5	40.6	40.6	18.7
Advanced Computer Architecture	35.2	24.1	31.5	22.2
Software Engineering	50.0	37.1	50.0	30.0
Mathematics of Data	29.7	18.9	32.4	24.3
ML for Physicists	54.8	51.2	52.4	42.9
Semiconductor Properties	59.1	31.8	31.8	50.0
Applied Data Analysis	35.7	42.9	35.7	32.1
Advanced General Chemistry	62.5	41.1	71.4	48.2
Information Processing and Communication	59.1	48.0	61.8	53.8
Analysis I	42.6	37.6	44.6	37.6
Average	44.8	35.8	43.2	34.4

Human Grader Remarks. During the human grading process, we asked the 28 graders to record their impressions of model answers. Overall, there was a general agreement that the model's responses were satisfactory for straightforward questions, but less so for those requiring logical reasoning or analysis. In the latter cases, it was noted that the model sometimes produced lengthy responses that added contextually relevant information but failed to actually solve the problem. In many instances, graders likened this behavior to students attempting to gain points by including all potentially relevant information related to a question's keywords. This behavior could also be an artifact of the prompting approach, however, as we used metacognitive and chain-of-thought prompting strategies to generate the outputs provided to human graders. While these strategies have the best performance on MCQ, they also tend to produce longer answers to open-ended questions.

Other issues identified include instances of factual inaccuracies (e.g., fabricated references) and contextual inaccuracies (e.g., using concepts unsuitable for the requested analysis). Finally, the model, at times, misunderstood the objective of the question (e.g., providing an implementation-specific answer when a student would instead interpret it as a design question). Regarding mathematical reasoning, apart from the previously mentioned limitations, the models struggled significantly with mathematical derivations requiring multiple steps, demonstrated a flawed understanding of imaginary numbers, and made errors in calculations.

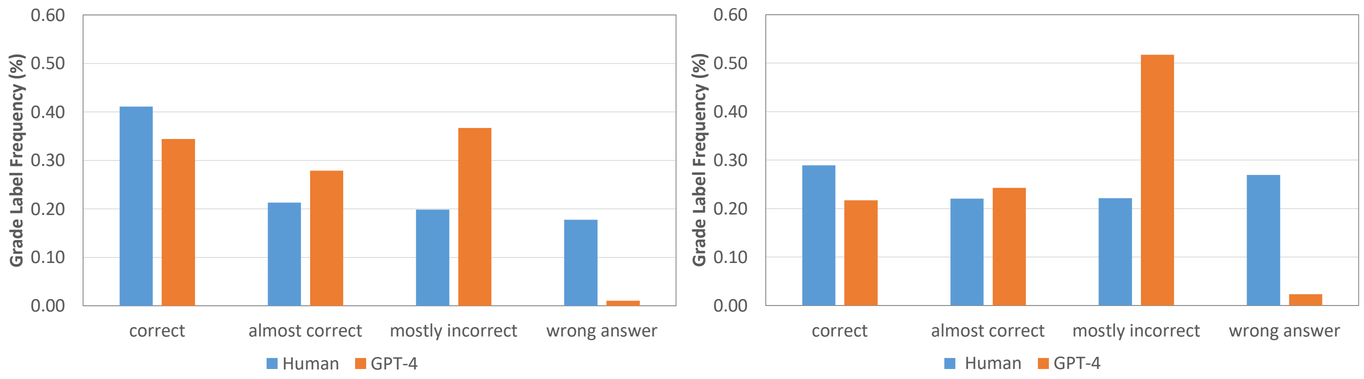

(a) GPT-4 Student

(b) GPT-3.5 Student

Fig. S1. Grade distribution. Distribution of grades assigned by our grader consortium (blue) and GPT-4 (orange) to responses provided by GPT-4 (left) and GPT-3.5 (right).

5. Additional Results

A. Individual Course Performance. Table S4 shows GPT-4 performance across all courses for open-answer questions. Table S5 shows GPT-4 performance across all courses for MCQ type of questions. Table S6 show GPT-3.5 performance across all courses for open-answer type of questions. Table S7 show GPT-3.5 performance across all courses for MCQ type of questions. As the exact course names are not important for this analysis, we anonymize course names when presenting results in these Tables.

Table S4. Performance of GPT-4 on open-answer questions for all courses categorized by prompting strategy. Majority corresponds to the performance of the majority vote aggregation strategy. Max corresponds to the maximum performance (the score when only one prompting strategy is required to return a correct answer for the model get the answer correct). Online courses typically have fewer open-answer questions as most evaluations in online courses are done through MCQA. * denotes required courses for a program (applies only for Bachelor and Master programs).

Course name	# questions	Zero-Shot	One-Shot	CoT (Zero-Shot)	CoT (Four-Shot)	Tree-of-Thought	Meta-cognitive	Expert	Self-Reflect	Majority	Max
*Biology #1	12	80.3	66.3	83.1	85.8	77.5	83.0	69.1	91.5	85.9	94.3
*Chemistry #1	74	69.9	69.9	79.9	80.0	75.4	80.9	75.4	82.7	80.9	94.0
*Computer Science #1	42	61.6	66.4	63.9	65.6	63.9	61.6	67.1	73.6	65.6	88.7
Computer Science #2	98	65.0	69.1	67.8	66.4	65.3	64.3	67.4	74.6	66.3	89.6
*Computer Science #3	42	64.8	67.2	75.2	78.3	67.9	64.8	74.3	75.9	70.3	93.5
*Computer Science #4	42	76.8	69.6	78.3	74.3	71.1	77.6	83.1	80.0	79.2	97.6
*Computer Science #5	223	68.5	69.7	74.3	71.5	68.6	70.9	73.4	73.4	73.1	89.4
*Computer Science #6	72	54.2	57.5	53.3	52.4	51.5	51.5	57.5	59.4	53.8	79.3
*Computer Science #7	70	81.1	82.1	85.9	88.8	80.2	84.1	88.4	89.4	87.4	98.1
Computer Science #8	36	57.1	62.6	70.0	65.4	53.3	58.0	59.8	70.0	57.1	89.7
*Computer Science #9	55	65.7	67.5	75.4	78.5	65.0	73.0	73.0	79.7	74.8	90.1
*Computer Science #10	34	82.1	80.1	86.1	83.1	84.1	78.2	89.1	90.1	87.1	98.0
*Data Science #1	28	71.1	67.5	72.3	71.1	69.8	65.1	66.3	72.3	68.7	90.3
Data Science #2	36	73.7	68.1	65.3	66.3	71.0	69.1	72.8	71.0	70.9	91.5
*Math #1	103	46.9	45.9	48.2	39.5	54.3	49.1	51.8	51.4	47.2	74.4
*Math #2	302	60.9	60.0	58.7	56.9	64.7	66.1	64.9	62.9	62.3	83.1
Online Life Sciences #1	9	36.7	40.4	66.2	70.1	58.9	59.0	58.9	62.7	58.9	92.6
Online Life Sciences #2	1	100.0	100.0	100.0	100.0	100.0	100.0	100.0	100.0	100.0	100.0
Online Life Sciences #3	1	0.0	0.0	33.0	33.0	33.0	33.0	66.0	33.0	33.0	66.0
Online Life Sciences #4	8	12.4	12.4	28.9	37.3	28.9	41.3	24.8	37.1	24.8	57.9
Online Life Sciences #5	7	61.7	57.0	66.4	80.7	71.3	71.1	61.7	66.4	61.7	85.4
Online Life Sciences #6	2	66.5	83.0	66.5	83.0	100.0	83.0	83.0	83.0	83.0	100.0
Online Life Sciences #7	3	66.3	22.0	55.3	33.0	22.0	22.0	44.0	22.0	22.0	88.7
Online Physics #1	3	55.3	66.0	100.0	100.0	77.7	100.0	77.3	100.0	100.0	100.0
Online Physics #2	2	66.5	49.5	83.0	33.0	66.5	100.0	83.0	83.0	66.5	100.0
Online Physics #3	7	66.4	37.9	61.4	47.3	71.0	71.0	66.3	75.9	61.6	75.9
Online Physics #4	4	83.0	58.3	49.8	33.0	58.0	91.5	74.8	58.0	66.5	91.5
Online Physics #5	3	66.3	22.0	55.3	55.3	66.3	66.3	55.3	55.3	55.3	77.3
Online Physics #6	13	48.4	40.8	68.8	45.8	56.0	63.7	58.6	63.7	61.2	71.4
Online Physics #7	12	46.8	41.3	44.0	38.5	46.9	60.7	46.8	49.5	41.3	80.2
Online Physics #8	4	58.0	41.3	49.5	66.3	41.3	49.5	49.8	58.0	49.5	74.8
Online Physics #9	7	18.9	9.4	28.3	37.7	37.7	37.7	28.3	33.0	33.0	47.1
Online Physics #10	27	75.1	77.6	86.3	88.8	66.4	87.5	87.5	88.8	86.3	97.5
*Physics #1	24	49.8	52.5	59.4	51.0	49.7	45.5	56.6	48.3	53.8	71.9
*Physics #2	45	58.2	64.9	66.3	57.5	52.2	53.7	73.1	57.4	56.0	87.2
Physics #3	3	33.0	33.0	33.0	33.0	55.0	33.0	44.0	44.0	33.0	66.0
Physics #4	24	37.2	33.0	51.0	46.8	49.6	41.3	46.9	48.3	44.1	63.5
*Physics #5	14	69.4	69.3	78.2	61.2	60.3	70.8	76.3	61.0	74.5	89.0
*Physics #6	68	66.4	69.7	68.8	68.3	69.3	74.3	66.8	77.7	71.3	90.5
*Physics #7	28	66.4	70.0	59.3	63.9	61.6	77.2	72.4	70.0	68.9	89.1
*Physics #8	53	46.2	46.2	53.7	51.2	40.5	45.5	49.3	57.5	46.8	71.3
*Physics #9	478	56.8	61.2	60.4	61.2	58.0	57.3	61.8	60.8	59.0	83.2

Table S5. Performance of GPT-4 on MCQs for all courses categorized by prompting strategy. Majority corresponds to the performance of the majority vote aggregation strategy. Max corresponds to the maximum performance (the score when only one prompting strategy is required to return a correct answer for the model get the answer correct). * denotes required courses for a program (applies only for Bachelor and Master programs).

Course name	# questions	Zero-Shot	One-Shot	CoT (Zero-Shot)	CoT (Four-Shot)	Tree-of-Thought	Meta-cognitive	Expert	Self-Reflect	Majority	Max
*Biology #1	48	62.5	75.0	81.3	83.3	81.3	79.2	56.3	83.3	85.4	87.5
Computer Science #2	54	31.5	35.2	46.3	42.6	35.2	35.2	27.8	38.9	42.6	59.3
*Computer Science #4	27	44.4	59.3	74.1	70.4	81.5	74.1	55.6	70.4	77.8	92.6
*Computer Science #5	20	60.0	80.0	90.0	90.0	85.0	80.0	55.0	75.0	90.0	100.0
Computer Science #8	229	49.3	55.0	58.1	64.9	57.6	60.7	51.1	53.7	66.8	85.2
*Computer Science #10	158	46.8	44.9	64.3	65.6	58.9	65.8	45.6	61.1	65.8	86.7
Computer Science #11	69	62.3	75.4	85.5	85.5	88.4	81.2	71.0	84.1	91.3	95.7
*Computer Science #12	36	36.1	33.3	75.0	80.6	63.9	63.9	30.6	80.6	77.8	94.4
Computer Science #13	60	48.3	56.7	65.0	65.0	61.0	63.3	51.7	73.3	65.0	88.3
*Computer Science #14	111	36.0	39.6	54.1	57.7	56.8	55.9	37.8	56.8	54.1	85.6
*Computer Science #15	676	56.4	67.6	78.1	79.9	77.1	77.8	58.7	76.9	82.1	94.8
Computer Science #16	41	48.8	63.4	80.5	87.8	73.2	80.5	63.4	73.2	85.4	95.1
*Math #1	118	19.5	16.1	34.7	39.8	27.1	34.7	21.2	38.1	35.6	61.0
*Math #2	31	29.0	32.3	38.7	32.3	54.8	38.7	29.0	35.5	41.9	87.1
Online Life Sciences #1	286	45.5	55.6	46.9	49.3	47.9	45.8	49.5	39.2	53.5	77.6
Online Life Sciences #2	33	63.6	78.8	78.8	72.7	72.7	78.8	66.7	72.7	81.8	87.9
Online Life Sciences #3	53	32.1	26.4	37.7	34.0	43.4	47.2	34.0	35.8	47.2	75.5
Online Life Sciences #4	226	43.4	54.9	47.8	48.2	49.6	54.4	45.6	56.6	51.3	81.4
Online Life Sciences #5	78	60.3	70.5	64.1	69.2	60.3	64.1	62.8	60.3	71.8	88.5
Online Life Sciences #6	85	54.1	62.4	60.0	61.2	58.8	57.6	58.8	57.6	60.0	82.4
Online Life Sciences #7	48	41.7	54.2	70.8	70.8	56.3	72.9	50.0	62.5	70.8	85.4
Online Life Sciences #8	156	78.2	90.4	87.8	87.8	86.5	85.9	82.7	83.3	93.6	98.7
Online Physics #1	90	65.6	68.9	73.3	66.7	66.7	67.8	57.8	72.2	73.3	92.2
Online Physics #2	70	61.4	72.9	71.4	77.1	75.7	74.3	58.6	74.3	78.6	94.3
Online Physics #3	74	50.0	56.8	47.3	50.0	44.6	52.7	46.6	56.8	56.8	81.1
Online Physics #4	40	50.0	57.5	52.5	55.0	45.0	60.0	37.5	50.0	50.0	82.5
Online Physics #5	32	37.5	56.3	68.8	53.1	68.8	56.3	34.4	65.6	68.8	93.8
Online Physics #6	55	45.5	58.2	41.8	56.4	40.0	45.5	45.5	50.9	50.9	80.0
Online Physics #7	33	63.6	60.6	57.6	54.5	60.6	69.7	63.6	57.6	66.7	84.8
Online Physics #8	111	48.6	61.3	61.3	57.7	61.3	65.8	54.9	61.3	61.3	84.7
Online Physics #9	60	48.3	53.3	63.3	65.0	61.7	61.7	63.3	65.0	65.0	90.0
Online Physics #10	51	39.2	50.9	43.1	41.2	54.9	52.9	47.1	64.7	56.9	80.4
Online Physics #11	107	59.8	70.1	69.2	71.9	69.2	67.3	52.3	60.7	74.8	86.0
Physics #3	58	53.4	55.2	65.5	67.2	58.6	60.3	51.7	69.0	69.0	89.7
Physics #4	36	44.4	41.7	50.0	55.6	52.8	58.3	38.9	55.6	69.4	88.9

Table S6. Performance of GPT-3.5 on open-answer questions for all courses categorized by prompting strategy. Majority corresponds to the performance of the majority vote aggregation strategy. Max corresponds to the maximum performance (the score when only one prompting strategy is required to return a correct answer for the model get the answer correct). Online courses typically have fewer open-answer questions as most evaluations in online courses are done through MCQA. * denotes required courses for a program (applies only for Bachelor and Master programs).

Course name	# questions	Zero-Shot	One-Shot	CoT (Zero-Shot)	CoT (Four-Shot)	Tree-of-Thought	Meta-cognitive	Expert	Self-Reflect	Majority	Max
*Biology #1	12	63.4	66.3	69.2	66.3	49.5	66.3	68.9	69.1	63.4	91.5
*Chemistry #1	74	60.1	62.7	61.9	65.5	65.0	62.8	59.6	66.5	65.1	86.3
*Computer Science #1	42	46.5	53.7	59.1	52.1	46.5	52.8	53.6	56.9	52.0	84.7
Computer Science #2	98	52.1	60.9	57.5	55.4	49.3	52.1	54.5	58.2	54.5	81.1
*Computer Science #3	42	59.2	48.0	60.7	63.2	55.1	58.5	65.6	60.8	55.2	83.1
*Computer Science #4	42	64.0	58.5	59.2	64.8	49.7	66.4	64.8	55.2	57.6	91.2
*Computer Science #5	223	58.2	60.6	56.2	57.6	51.3	59.5	59.5	57.7	58.9	81.8
*Computer Science #6	72	40.9	43.2	41.3	35.8	37.2	38.1	40.4	39.0	39.0	62.6
*Computer Science #7	70	76.4	72.5	72.9	76.3	66.3	73.0	72.9	78.8	74.0	93.7
Computer Science #8	36	58.9	54.4	55.2	57.0	34.9	46.9	57.1	48.7	51.5	75.7
*Computer Science #9	55	56.0	66.3	69.3	70.0	60.1	62.0	65.1	70.6	63.9	87.1
*Computer Science #10	34	69.4	69.3	69.3	70.4	54.6	62.5	71.3	69.3	65.4	91.1
*Data Science #1	28	54.4	59.2	57.9	56.8	54.4	55.6	67.5	54.5	59.2	82.0
Data Science #2	36	49.6	48.7	56.1	47.7	48.8	56.1	45.9	45.9	43.1	79.3
*Math #1	103	40.8	39.8	40.8	41.1	41.7	43.3	42.4	39.5	41.1	62.8
*Math #2	302	50.4	50.4	50.4	43.9	47.2	55.7	52.3	48.9	51.9	72.6
Online Life Sciences #1	9	33.1	47.8	47.7	66.4	47.9	58.9	62.6	70.1	55.2	92.6
Online Life Sciences #2	1	66.0	66.0	100.0	33.0	66.0	66.0	66.0	33.0	66.0	100.0
Online Life Sciences #3	1	0.0	0.0	33.0	33.0	33.0	33.0	33.0	33.0	33.0	33.0
Online Life Sciences #4	8	37.3	37.3	41.3	37.1	41.4	24.8	24.8	33.0	37.1	66.4
Online Life Sciences #5	7	28.3	52.3	52.1	37.9	47.3	37.9	52.1	56.7	47.4	71.1
Online Life Sciences #6	2	100.0	100.0	100.0	100.0	66.5	83.0	83.0	100.0	100.0	100.0
Online Life Sciences #7	3	22.0	44.3	55.3	33.0	66.3	33.3	55.3	55.3	55.3	66.3
Online Physics #1	3	55.3	44.0	77.7	100.0	44.0	100.0	100.0	77.7	100.0	100.0
Online Physics #2	2	49.5	83.0	83.0	66.5	66.5	83.0	66.0	49.5	49.5	100.0
Online Physics #3	7	52.1	52.0	52.0	47.3	56.7	33.0	37.7	47.1	42.4	71.1
Online Physics #4	4	58.0	58.0	49.8	33.0	33.0	49.5	49.5	41.5	49.8	66.3
Online Physics #5	3	33.0	22.0	44.3	55.3	33.0	22.0	66.3	55.3	22.0	88.7
Online Physics #6	13	43.2	38.1	50.9	43.2	40.7	45.8	58.6	40.7	45.8	68.9
Online Physics #7	12	41.3	46.9	38.5	38.5	44.0	41.3	38.6	52.4	38.5	71.8
Online Physics #8	4	41.5	33.0	58.0	49.5	49.5	41.3	49.8	58.0	49.5	66.5
Online Physics #9	7	33.0	18.9	37.7	28.3	33.0	18.9	37.7	42.4	37.7	51.9
Online Physics #10	27	68.9	62.8	73.9	87.6	59.0	76.3	80.0	80.1	77.7	96.2
*Physics #1	24	30.3	38.6	33.0	37.2	23.4	33.0	31.7	28.9	35.8	51.0
*Physics #2	45	43.4	47.8	39.7	42.6	36.7	36.7	41.9	39.7	37.5	65.6
Physics #3	3	33.0	33.0	44.0	22.0	44.0	44.0	33.0	44.0	33.0	66.0
Physics #4	24	35.9	33.0	30.3	35.8	39.9	38.5	37.2	38.5	35.8	56.5
*Physics #5	14	57.1	40.0	50.0	64.2	38.6	43.4	50.1	63.8	52.0	79.7
*Physics #6	68	59.4	57.6	59.0	49.1	48.6	54.5	54.6	54.6	54.5	77.7
*Physics #7	28	58.1	62.8	50.9	48.5	45.0	56.9	54.5	43.7	54.5	74.7
*Physics #8	53	38.6	43.0	38.6	38.0	34.9	39.9	37.4	36.8	37.4	58.1
*Physics #9	478	43.2	45.5	42.5	42.5	40.0	42.7	44.6	42.6	42.0	63.7

Table S7. Performance of GPT-3.5 on MCQs for all courses categorized by prompting strategy. Majority corresponds to the performance of the majority vote aggregation strategy. Max corresponds to the maximum performance (the score when only one prompting strategy is required to return a correct answer for the model get the answer correct). * denotes required courses for a program (applies only for Bachelor and Master programs).

Course name	# questions	Zero-Shot	One-Shot	CoT (Zero-Shot)	CoT (Four-Shot)	Tree-of-Thought	Meta-cognitive	Expert	Self-Reflect	Majority	Max
*Biology #1	48	39.6	50.0	45.8	54.2	56.2	41.7	50.0	43.8	56.2	79.2
Computer Science #2	54	22.2	25.9	24.1	33.3	27.8	31.5	29.6	24.1	31.5	61.1
*Computer Science #4	27	37.0	48.1	37.0	44.4	51.9	44.4	48.1	48.1	55.6	77.8
*Computer Science #5	20	70.0	65.0	65.0	60.0	50.0	65.0	60.0	50.0	70.0	90.0
Computer Science #8	229	47.6	48.0	38.4	43.2	33.2	43.2	46.7	36.2	52.0	88.2
*Computer Science #10	158	37.9	30.4	37.6	45.9	40.5	40.5	37.3	39.5	44.3	80.4
Computer Science #11	69	59.4	63.8	63.8	69.6	53.6	65.2	62.3	60.9	71.0	89.9
*Computer Science #12	36	44.4	36.1	47.2	47.2	25.0	27.8	27.8	41.7	50.0	86.1
Computer Science #13	60	25.0	28.3	38.3	50.0	33.3	46.7	31.7	43.3	45.0	76.7
*Computer Science #14	111	43.2	30.6	42.3	49.5	41.8	40.5	36.0	30.6	42.3	87.4
*Computer Science #15	676	51.3	55.6	53.4	55.9	43.8	54.9	52.9	52.2	62.1	90.1
Computer Science #16	41	36.6	63.4	48.8	51.2	34.1	51.2	48.8	51.2	63.4	82.9
*Math #1	118	14.4	14.4	15.3	19.5	9.4	18.6	13.6	10.2	19.5	38.1
*Math #2	31	19.4	32.3	41.9	29.0	32.3	38.7	32.3	32.3	32.3	83.9
Online Life Sciences #1	286	45.5	48.6	44.1	45.1	38.6	47.9	48.3	42.7	51.0	81.5
Online Life Sciences #2	33	69.7	66.7	78.8	60.6	78.8	66.7	69.7	69.7	78.8	93.9
Online Life Sciences #3	53	22.6	28.3	24.5	32.1	39.6	33.9	39.6	28.3	35.8	77.4
Online Life Sciences #4	226	48.2	50.0	48.2	42.9	44.7	47.8	47.3	46.5	55.3	83.2
Online Life Sciences #5	78	58.9	61.5	66.7	52.6	47.4	61.5	55.1	57.7	69.2	84.6
Online Life Sciences #6	85	48.2	57.6	58.8	57.6	54.1	67.1	56.5	52.9	61.2	88.2
Online Life Sciences #7	48	56.2	39.6	52.1	45.8	50.0	50.0	52.1	50.0	68.8	87.5
Online Life Sciences #8	156	66.0	75.6	73.1	69.9	63.5	69.2	69.2	60.9	80.8	93.6
Online Physics #1	90	52.2	52.2	55.6	52.2	51.7	53.3	51.1	52.2	56.7	84.4
Online Physics #2	70	52.9	60.0	48.6	52.9	47.1	62.9	54.3	41.4	62.9	90.0
Online Physics #3	74	44.6	44.6	40.5	27.0	40.5	55.4	48.6	41.9	45.9	81.1
Online Physics #4	40	42.5	32.5	40.0	45.0	27.5	50.0	42.5	41.0	52.5	77.5
Online Physics #5	32	25.0	25.0	43.8	37.5	37.5	18.8	43.8	43.8	40.6	84.4
Online Physics #6	55	50.9	41.8	34.5	34.5	34.5	47.3	43.6	37.0	50.9	89.1
Online Physics #7	33	51.5	42.4	56.3	51.5	39.4	63.6	57.6	51.5	60.6	87.9
Online Physics #8	111	48.6	49.5	50.5	45.9	36.0	44.1	50.5	47.7	52.3	80.2
Online Physics #9	60	50.0	58.3	53.3	53.3	50.0	61.7	43.3	48.3	60.0	88.3
Online Physics #10	51	52.9	47.1	49.0	43.1	41.2	43.1	43.1	49.0	58.8	76.5
Online Physics #11	107	45.8	57.9	52.3	56.1	45.8	59.8	48.6	47.7	58.9	89.7
Physics #3	58	39.7	46.6	50.9	27.6	44.8	51.7	37.9	41.4	46.6	87.9
Physics #4	36	47.2	38.9	33.3	50.0	36.1	47.2	44.4	33.3	50.0	88.9

B. Impact of Prompting Strategy. Table S8 shows the average GPT-4 and GPT-3.5 performance for each prompting strategy. GPT-4 outperforms GPT-3.5 across all prompting strategies. When answering MCQs, four-shot CoT (5) emerges as GPT-4's best-performing strategy, while zero-shot achieves the lowest performance. Curiously, the same ranking does not transfer to open-answer questions, where self-reflect (9) emerges as the best strategy, followed by Expert Prompting (4). Zero-shot prompting remains the least performant. However, based on a survey of reports submitted by Masters students for a class project in a Natural Language Processing (NLP) course, we found students to be most likely to use Zero-shot, Expert, and Zero-shot COT prompting, as these are the most intuitive strategies and the ones that require the least amount of preparation work.

Table S8. Performance of GPT-3.5 and GPT-4 on all MCQs and open-answer questions, categorized by prompting strategy. The most effective prompting strategy for each model is underlined. Open-answer questions are graded by GPT-4. All scores are provided with 95% confidence intervals.

Question Type	Model	Zero-Shot	One-Shot	CoT (Zero-Shot)	CoT (Four-Shot)	Tree-of-Thought	Meta-cognitive	Expert	Self-Reflect
MCQ	GPT-3.5	46.4 ± 1.7	48.5 ± 1.7	47.8 ± 1.7	48.3 ± 1.7	42.1 ± 1.7	49.8 ± 1.7	47.6 ± 1.7	45.0 ± 1.7
	GPT-4	50.1 ± 1.6	58.7 ± 1.6	63.2 ± 1.6	64.8 ± 1.6	62.1 ± 1.6	63.8 ± 1.6	52.2 ± 1.6	62.5 ± 1.6
Open Answer	GPT-3.5	52.7 ± 1.4	53.9 ± 1.4	53.8 ± 1.4	52.5 ± 1.4	48.4 ± 1.3	53.9 ± 1.4	54.5 ± 1.4	53.5 ± 1.4
	GPT-4	62.8 ± 1.5	62.9 ± 1.5	66.4 ± 1.4	64.3 ± 1.5	63.9 ± 1.4	65.9 ± 1.4	67.6 ± 1.4	69.1 ± 1.4

C. Performance by Language. In our dataset, we have 70.5% of English questions and 29.5% of French questions. Table S9 shows performance by language across models and question types. Table S10 shows GPT-4 performance per language per prompting strategy across all question types.

Table S9. Performance of GPT-3.5 and GPT-4 on MCQs and open-answer questions, categorized by the question language. Open-answer questions are graded by GPT-4. Performance is presented with 95% confidence interval.

Question Type	Model	English	French
MCQ	GPT-3.5	47.3 ± 1.7	32.5 ± 4.9
	GPT-4	63.2 ± 1.7	48.0 ± 5.1
Open Answer	GPT-3.5	52.1 ± 1.9	54.5 ± 2.0
	GPT-4	65.4 ± 2.0	67.3 ± 2.0

Table S10. Performance of GPT-4 per language on MCQs and open-answer questions, categorized by prompting strategy. The most effective prompting strategy for each language is underlined. Open-answer questions are graded by GPT-4. All scores are provided with 95% confidence intervals.

Language	Question Type	Zero-Shot	One-Shot	CoT (Zero-Shot)	CoT (Four-Shot)	Tree-of-Thought	Meta-cognitive	Expert	Self-Reflect
English	MCQ	51.8 ± 1.7	60.4 ± 1.7	64.4 ± 1.7	66.0 ± 1.7	63.2 ± 1.7	65.0 ± 1.7	53.4 ± 1.7	63.3 ± 1.7
	Open Answer	62.4 ± 2.1	62.5 ± 2.1	67.4 ± 2.0	65.7 ± 2.0	61.8 ± 2.0	63.2 ± 2.0	67.7 ± 2.0	70.0 ± 2.0
French	MCQ	37.4 ± 5.3	42.6 ± 5.4	51.8 ± 5.4	53.3 ± 5.4	51.9 ± 5.4	52.3 ± 5.3	40.7 ± 5.4	55.7 ± 5.4
	Open Answer	63.1 ± 2.1	63.1 ± 2.1	65.3 ± 2.0	63.1 ± 2.1	65.7 ± 2.0	68.4 ± 2.0	67.5 ± 2.0	68.4 ± 2.1

Impact of Prompt Language. Tables S9 and S10 show differing performance on English questions compared to French questions. Unfortunately, the subsets of courses in our dataset in English and French mostly does not intersect, precluding a conclusive comparison between these performance measurements. However, given that AI assistants are often predominantly trained on English text data, these results raise a question of whether performance on French questions could be increased further, through creative cross-lingual prompting.

Consequently, we explore whether a student user could achieve better performance by varying the language of the prompting instruction. We employ three variations of the metacognitive prompting strategy (which ranks among the top-performing strategies), where we vary the language and the wording of the instruction and the question, as schematized in Figure S2: *Vanilla*, *Language-inverted*, and *Guided*. In the *Vanilla* setting, we provide the prompt instruction and question in the same language. In the *Inverted* setting, we provide the instruction and question in different languages. Finally, in the *Guided* setting, we provide the instruction and question in different languages but clarify in the instruction that the answer should be provided in the same language as the question. We focus on MCQ-based performance to avoid potential language bias from GPT-4 as a grader, assessing the impact of these three variations across all English and French MCQs of our dataset.

Table S11. Performance comparison of GPT-3.5 and GPT-4 across the three different prompting strategies (*vanilla*, *language inverted*, and *guided*), categorized by question language. The most effective prompting strategy for each language is underlined. All scores are provided with 95% confidence interval.

Question Language	Vanilla		Inverted		Guided	
	GPT-3.5	GPT-4	GPT-3.5	GPT-4	GPT-3.5	GPT-4
English	51.2 ± 1.8	**65.4** ± 1.5	44.5 ± 1.7	61.7 ± 1.6	46.8 ± 1.7	61.4 ± 1.6
French	39.4 ± 4.2	**53.1** ± 4.3	38.3 ± 4.1	51.9 ± 4.4	36.2 ± 4.0	53.0 ± 4.1

As illustrated in Table S11, the average scores for the *Vanilla* setting are higher for both English and French compared to the *language-inverted* setting, indicating that instructing the model in the same language as the question leads to higher performance for both models compared to when the question is in a different language. Finally, guiding the model by asking it to reason and answer in the same language as the question, even if the instructions are in another language (i.e., the *guided* setting), enhances the performance for GPT-4 on French questions, yielding a score equivalent to providing instructions in French. Taken together, our results show that there is little benefit from prompting the model in English (a language that most pretrained models have likely seen more data from) compared to the language of the question.

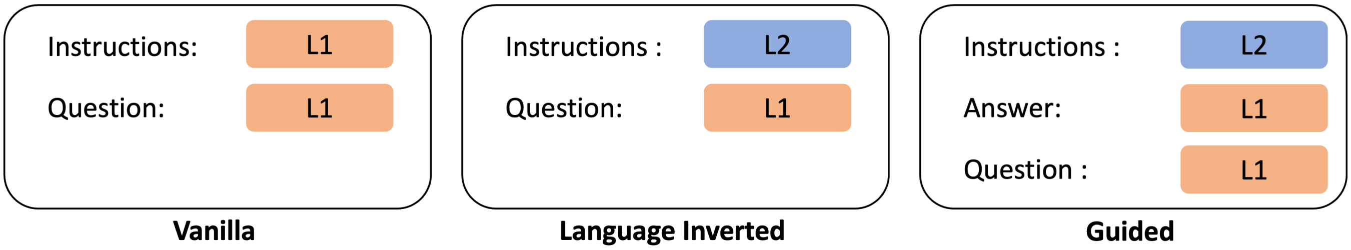

Fig. S2. The three language-related prompting strategies. Given two languages *L1* and *L2*, and a question in language *L1*, (1) **Vanilla**: provides instructions in *L1*; (2) **Language Inverted**: provides instructions in *L2*; (3) **Guided**: provides instructions in *L2*, specifying that the question is in *L1*, and that it should be answered in *L1* as well.

References

1. T Brown, et al., Language models are few-shot learners in *Advances in Neural Information Processing Systems*, eds. H Larochelle, M Ranzato, R Hadsell, M Balcan, H Lin. (Curran Associates, Inc.), Vol. 33, pp. 1877–1901 (2020).
2. N Reimers, I Gurevych, Sentence-bert: Sentence embeddings using siamese bert-networks in *Proceedings of the 2019 Conference on Empirical Methods in Natural Language Processing*. (Association for Computational Linguistics), (2019).
3. Y Liu, et al., Roberta: A robustly optimized bert pretraining approach (2019).
4. B Xu, et al., Expertprompting: Instructing large language models to be distinguished experts (2023).
5. J Wei, et al., Chain-of-thought prompting elicits reasoning in large language models (2023).
6. T Kojima, SS Gu, M Reid, Y Matsuo, Y Iwasawa, Large language models are zero-shot reasoners. *ArXiv* **abs/2205.11916** (2022).
7. S Yao, et al., Tree of thoughts: Deliberate problem solving with large language models (2023).
8. R Wang, et al., Self-critique prompting with large language models for inductive instructions (2023).
9. A Madaan, et al., Self-refine: Iterative refinement with self-feedback. *arXiv:2303.17651* (2023).
10. Y Wang, Y Zhao, Metacognitive prompting improves understanding in large language models (2023).
11. SC Karpen, AC Welch, Assessing the inter-rater reliability and accuracy of pharmacy faculty's bloom's taxonomy classifications. *Curr. Pharm. Teach. Learn.* **8**, 885–888 (2016).
